# Topological charge of soft X-ray vortex beam determined by inline holography

**DOI:** 10.1038/s41598-022-04933-5

**Published:** 2022-01-20

**Authors:** Yuta Ishii, Hironori Nakao, Masaichiro Mizumaki, Yusuke Wakabayashi, Taka-hisa Arima, Yuichi Yamasaki

**Affiliations:** 1 Department of Physics, Tohoku University, Sendai, 980-8578 Japan; 2 Photon Factory, Institute of Materials Structure Science, High Energy Accelerator Research Organization, Tsukuba, 305-0801 Japan; 3 Japan Synchrotron Radiation Research Institute (JASRI/SPring-8), Sayo, 679-5198 Japan; 4 RIKEN Center for Emergent Matter Science (CEMS), Wako, 351-0198 Japan; 5 Research and Services Division of Materials Data and Integrated System (MaDIS), National Institute for Materials Science (NIMS), Tsukuba, 305-0047 Japan

**Keywords:** X-rays, Imaging and sensing, Microscopy

## Abstract

A Laguerre–Gaussian (LG) vortex beam having a spiral wavefront can be characterized by its topological charge (TC). The TC gives the number of times that the beam phase passes through the interval $[0, 2\pi ]$ following a closed loop surrounding the propagation axis. Here, the TC spectra of soft X-ray vortex beams are acquired using the in-line holography technique, where interference between vortex waves produced from a fork grating and divergent waves from a Fresnel zone plate is observed as a holographic image. The analyses revealed the phase distributions and the TC for the LG vortex waves, which reflects topological number of the fork gratings, as well as for the Hermite–Gaussian (HG) mode waves generated from the other gratings. We also conducted a simulation of the present technique for pair annihilation of topological defects in a magnetic texture. These results may pave the way for development of probes capable of characterizing the topological numbers of magnetic defects.

## Introduction

Soft X-ray vortex beams, which have a spiral wavefront and finite orbital angular momentum, are expected to provide new tools for probing and/or manipulating the magnetic properties of materials, because of the high sensitivity of soft X-rays to the spin density of unoccupied states. Visible-light vortex beams have attracted intensive interest for decades^1^, because of their various potential applications, as for example, optical tweezers^2^ and super-resolution microscopy^3^ as well as the physics of optical induced excited states in magnetic materials^4–6^. To bring new techniques to X-ray measurements, generation of X-ray vortex beams has been also investigated using undulators, spiral zone plates, and diffractive optics^7–14^.

A possible practical application of soft X-ray vortex beams is as a probe for magnetic topological defects. Recently soft X-ray plane waves were reported to be transformed into vortex waves via an artificial spin lattice containing a topological defect^15^. Similarly, soft X-ray vortex beams are expected to be generated via topological defects in magnetic materials, such as magnetic Bloch points in helical or skyrmion lattices^16–20^. These defects, especially their dynamics, have attracted much attention because of their potential applications in magnetic memory devices^21,22^. Notably, the topological number of these defects, which reflects the winding number on the magnetic texture, can be transcribed to that of the vortex waves. Thus, detecting vortex beams generated from a topological defect would lead to effective magnetic probes for characterizing the topological features of magnetic textures. To this end, visualizing the spiral wavefront of a soft X-ray vortex beam and determining its topological number are critical, yet no technique has been established for this purpose.

**Figure 1:**
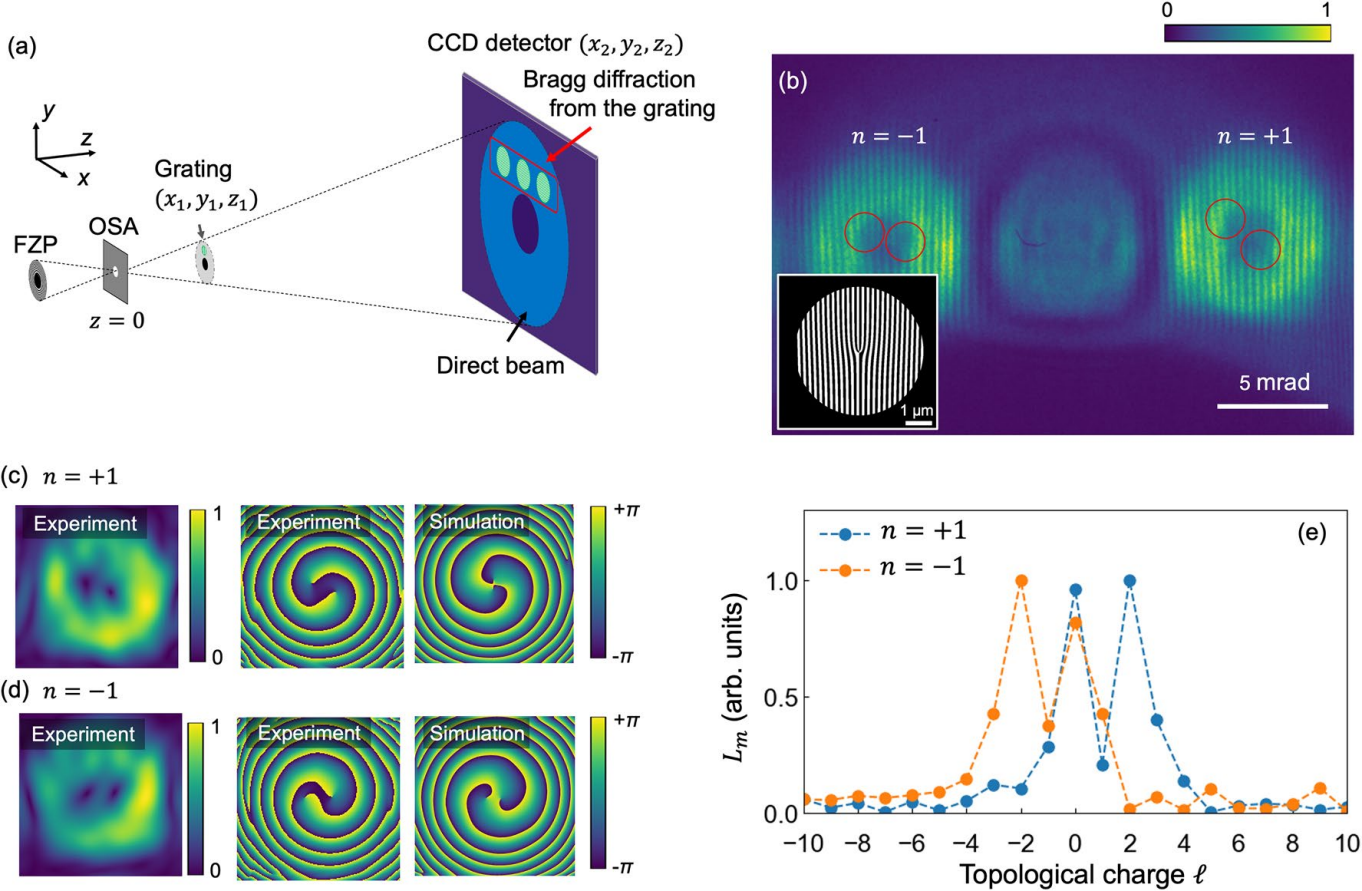
(**a**) Schematic of the experimental setup for soft X-ray inline holography. Incident X-rays are focused via a Fresnel zone plate (FZP). The first-order diffraction from the FZP is selected by an order sorting aperture (OSA) placed in the vicinity of the focal point. A grating placed downstream of the focal point produces Bragg diffraction waves, which interfere with direct waves transmitting outside the grating. The interference pattern is observed at the CCD detector as a holographic image. (**b**) Hologram pattern from the fork grating with $b = 2$ shown in the inset. Red circles indicate fork shaped patterns. (**c**,**d**) $A(\rho _{fn}, \phi _{fn})$ (first column) and phase distribution (second column) of the vortex diffraction waves for (**b**) $n = +1$ and (**c**) $n = -1$, as obtained using spatial frequency filtering (refer to the main text). Figures in third column present simulated phase patterns for these vortex diffraction waves. (**e**) TC spectra for the $nb = \pm \,2$ diffraction waves obtained using the method described in the main text.

Vortex waves can be expressed as a Laguerre–Gaussian (LG) mode^23^ with an azimuthal wave function $\exp (i\ell \phi )$, where $\phi$ is the azimuthal angle in cylindrical coordinates. The parameter $\ell$ is the topological number, which is called the topological charge (TC); it represents the number of times that the beam phase passes through the interval $[0,2\pi ]$ following a closed loop *C*. It is given by

1 $$\begin{aligned} \ell = \frac{1}{2\pi } \oint _C \nabla \theta ({\varvec{r}}) \cdot d{\varvec{r}}, \end{aligned}$$

where $\theta$ represents the phase distribution of the wave. In our recent study, inline holography experiment (details of this technique are found in the second section) was performed to visualize a single spiral phase distribution of soft X-ray vortex beam produced from a fork grating (such as that shown in the inset of Fig. 1b) with the topological number $b = 1$^24^. Meanwhile, it is crucial for practical application of magnetic materials research to determine the exact TC of vortex waves from the phase distribution, and to apply this technique to other gratings, such as that with topological number higher than $b = 1$.

In the present study, we successfully acquired the TC spectra for various soft X-ray vortex waves generated from several types of gratings by using the inline holography technique. The present technique was also applied to a Hermite–Gauss (HG) X-ray mode, which can be used to produce LG and arbitrary polarization vortex modes^23^. In addition, we present a numerical simulation of inline holography for pair annihilation of topological defects in a helical magnetic lattice. It demonstrates that acquiring the TC spectra of generated vortex waves is an effective approach to investigate the dynamics of topological defects.

## Inline holography experiment and results

The experimental setup for inline holography is illustrated in Fig. 1a. Incident X-rays are first focused by a Fresnel zone plate (FZP), and the first-order diffraction waves from the FZP are selected by an order sorting aperture (OSA) placed at the focal point. Vortex waves generated as Bragg diffractions from the grating, which is positioned downstream of the focal point, interferes with the direct-beam waves from the FZP transmitting outside the grating. Recording the resultant interference patterns enables direct acquisition of phase information for the vortex waves.

The transmission of a fork grating with topological number *b* is expressed as^13^

2 $$\begin{aligned} t(\rho ,\phi ) =&\frac{1}{2} \left( 1+ \mathrm{sgn}\left[ \mathrm{sin}\left( \frac{2\pi }{d} \rho \mathrm{cos}\phi + b\phi \right) \right] \right) , \end{aligned}$$

in two-dimensional polar coordinates ($\rho , \phi$), where *d* is the pitch of the grating far from the center. The vortex beam with $\ell = nb$ is produced as the *n*-th Bragg diffraction from this grating. Here we performed inline holography for a fork grating with $b = 2$, which is illustrated in the inset of Fig. 1b. The LG states of *n*-th order Bragg diffraction from this fork grating can be ideally characterized by $\ell = nb = 2n$. Figure 1b presents the interference pattern for $n = \pm 1$ diffraction waves from the fork grating positioned at $z_1 = 600\ \upmu$m from the focal point and on the up-side of the annular transmitting waves from the FZP. The Holographic image was obtained using an CCD camera at $$z_2 = 280\ $$mm. Intensity modulation is observed along the horizontal direction, and the upper half of the diffraction pattern contains two additional stripes compared with the lower half, resulting in the appearance of two fork-shaped patterns around the center.

We used spatial frequency filtering to extract the phase distribution of these diffraction waves (details of the method are available in Ref.^24^). The intensity fo *n*th Bragg diffraction from a fork grating is calculated as^24^

3 $$\begin{aligned} I^{\text {inter}}_{nb} \propto |{\mathscr {J}}^\prime _{nb}| \sin \left\{ kR_2+nb\phi _{fn} + \alpha _{nb} + n(b-1)\frac{\pi }{2}\right\} , \end{aligned}$$

with

4 $$\begin{aligned}&{\mathscr {J}}^\prime _{nb}(\rho _{fn}) = \int _0^a e^{if_0\rho ^2} \rho J_{nb}(\rho _{fn}\rho ) d\rho \equiv |{\mathscr {J}}_{nb}^\prime (\rho _{fn})|e^{i\alpha _{nb}}, \end{aligned}$$

where ($\rho _{fn}$, $\phi _{fn}$) are the local cylindrical coordinates with the center of the *n*th Bragg diffraction as the origin, and $J_{nb}$ is the *nb*-th Bessel function of the first kind. The parameter $R_2$ represents the phase difference between the reference and diffraction waves and is expressed as

5 $$\begin{aligned} R_2=\frac{z_1}{2z_2(z_2-z_1)}\left\{ \left( x_2-\frac{z_2}{z_1}x_g\right) ^2+\left( y_2-\frac{z_2}{z_1}y_g\right) ^2\right\} , \end{aligned}$$

with the center of the grating $(x_g, y_g, z_1)$ and the detector plane $(x_2, y_2, z_2)$. The Fourier transform ${\mathscr {F}}$ of the interference intensity is expressed as

6 $$\begin{aligned} {\mathscr {F}}(I) = G_{\mathrm{DC}} + G_{\mathrm{AC+}} + G_{\mathrm{AC-}}, \end{aligned}$$

where $G_{\mathrm{DC}}$ and $G_{\mathrm{AC\pm }}$ are the DC and AC components of ${\mathscr {F}}(I)$, respectively. An interference term is included in $G_{\mathrm{AC\pm }}$. Thus, the spiral phase distribution can be extracted by filtering $G_{\mathrm{AC+}}$, applying an inverse Fourier transform ${\mathscr {F}}^{-1}$, and multiplying by the term $\exp (-ikR_2)$:

7 $$\begin{aligned} f(\rho _{fn}, \phi _{fn})&\equiv \exp (-ikR_2){\mathscr {F}}^{-1}(G_{\mathrm{AC+}}) \end{aligned}$$

8 $$\begin{aligned}= A(\rho _{fn}, \phi _{fn}) \exp (inb\phi _{fn} + i\alpha _{nb}), \end{aligned}$$

where $A(\rho _{fn}, \phi _{fn})$ is the amplitude of the vortex wave.

$A(\rho _{fn}, \phi _{fn})$ and the phase distributions for the $nb = \pm 2$ diffraction waves are presented in the figures in the first and second columns of Fig. 1c,d. A double spiral phase distribution is obtained for each diffraction wave, and the angular rotation direction is reversed between them. In addition, Fermat’s spiral-like patterns are observed, arising from the term of $\exp (i\alpha _{nb})$^24^. Each phase pattern contains two singularities near the center, which indicates that a vortex beam with $\ell = 2$ splits into two vortex beams with $\ell = 1$. This phenomenon, known as charge splitting, is attributed to contamination of the $\ell = 0$ mode as a result of imperfection in the shape of the fork grating^25^. We also simulated the phase distribution for these waves using the scaled fast Fourier transform (FFT) method^26^. In this calculation, we assumed an ideal FZP and fork grating. The figures in the third column of Fig. 1c,d show the simulated phase patterns, which reasonably reproduce the experimental results.

To extract the TC of the vortex waves, we calculated $L_m$ expressed as

9 $$\begin{aligned} L_m \equiv \left| \int _0^{\rho _{\mathrm{max}}} \rho \mathrm{d}\rho \int _0^ {2\pi } \mathrm{d}\phi f(\rho ,\phi ) \exp (-im\phi ) \right| , \end{aligned}$$

where $\rho _{\mathrm{max}}$ reflects the size of the region which we took as the diffraction image (as shown in Fig. 1c,d). $L_m$ represents the decomposition to the $\ell = m$ mode of a vortex wave and thus gives the TC spectrum amplitudes. $L_m$ includes the integral of $A(\rho _{fn}, \phi _{fn})$, which indicates that $L_m$ is ideally independent of $\rho _{\mathrm{max}}$ if $\rho _{\mathrm{max}}$ is sufficiently large to include the diffraction peak, because $A(\rho _{fn}, \phi _{fn})$ is ideally zero outside the diffraction region. Figure 1e shows the TC spectrum obtained for the $n = \pm 1$ diffraction waves. This spectrum shows well-defined peaks at $\ell = \pm 2$ for the $n = \pm 1$. In addition, an additional strong peak appears at $\ell = 0$ for both waves, clearly indicating contamination of the $\ell = 0$ mode.

**Figure 2:**
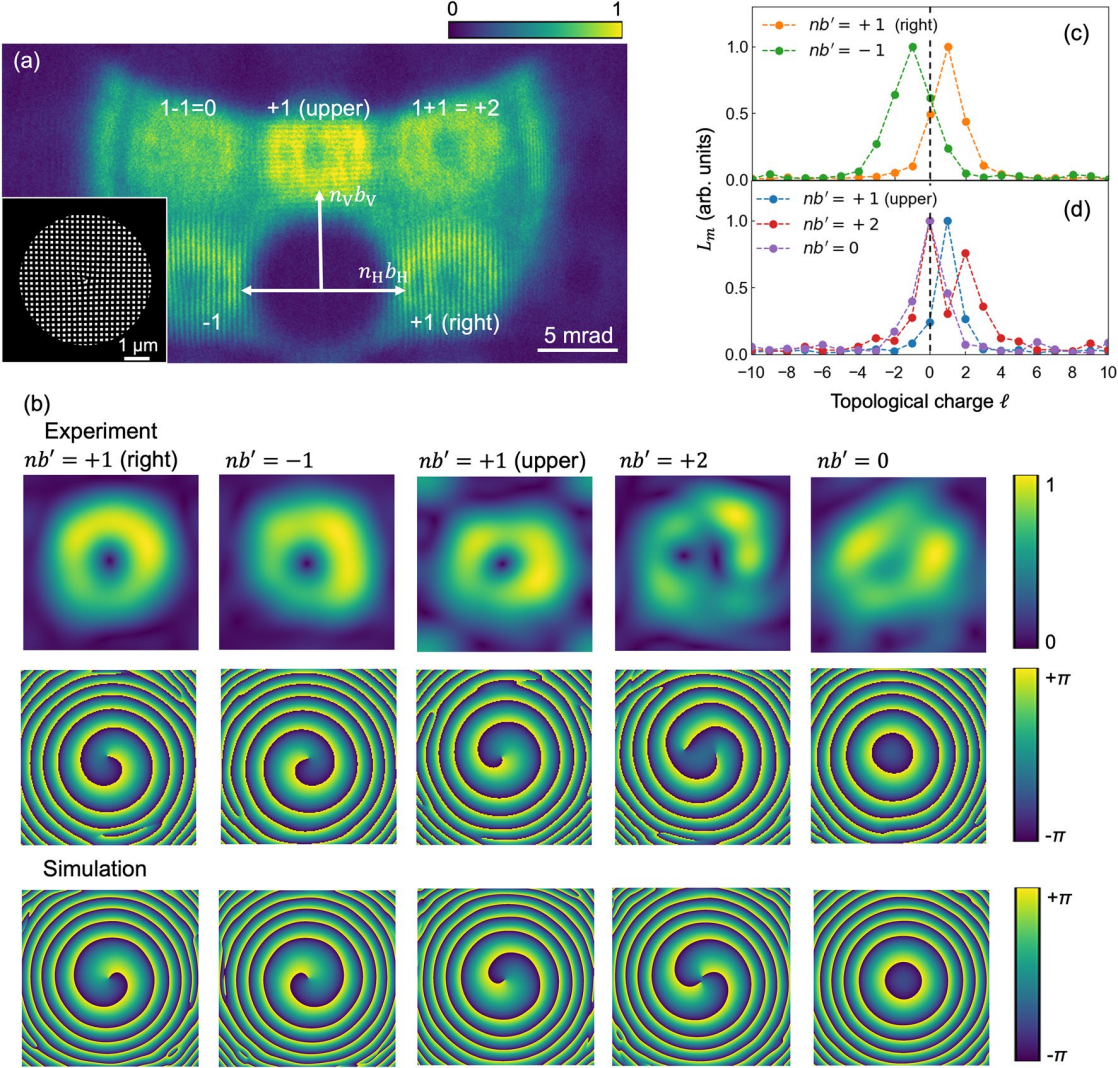
(**a**) Hologram pattern from the fork grating with $b_{\mathrm{H}}$ = 1 and $b_{\mathrm{V}}$ = 1. The inset shows a schematic of the fork grating. (**b**) $A(\rho _{fn}, \phi _{fn})$ (first row) and phase distribution (second row) of the vortex diffraction waves for $nb^{\prime } = n_{\mathrm{H}} b_{\mathrm{H}} +n_{\mathrm{V}} b_{\mathrm{V}} =$ 0, $\pm 1$, $+2$. Images in the third row present simulated phase patterns for these vortex diffraction waves. (**c**,**d**) TC spectrum for each diffraction wave.

We also performed in-line holography for another fork grating constructed from two perpendicularly oriented *b* = 1 fork gratings, as illustrated in the inset of Fig. 2a. The transmission of this fork grating is given by

10 $$\begin{aligned} t(\rho ,\phi ) =&\frac{1}{2} \left( 1+ \mathrm{sgn}\left[ \mathrm{sin}\left( \frac{2\pi }{d} \rho \mathrm{cos}\phi + b_H\phi \right) \right] \right) \nonumber \\&\times \frac{1}{2}\left( 1+ \mathrm{sgn}\left[ \mathrm{sin}\left( \frac{2\pi }{d} \rho \mathrm{sin}\phi + b_V\phi \right) \right] \right) , \end{aligned}$$

with topological numbers of two perpendicular gratings $b_H =1$ and $b_V = 1$. This grating produces vortex waves as Bragg diffraction waves distributed over a square lattice. These diffraction waves are ideally expressed by a TC described as $\ell = nb^\prime = n_Hb_H + n_Vb_V$, where $n_H$ and $n_V$ are the order of the Bragg diffraction along the horizontal and vertical directions, respectively; in one example, $(n_H, n_V) = (+1, +1)$ corresponds the $\ell = +2$ state. Figure 2a shows the interference diffraction pattern from the fork grating, which is placed at $z_1 = 600\ \upmu$m from the focal point and on the downside of the annular transmitting waves from the FZP. For $(n_H, n_V) = (\pm 1, 0)$ and $(n_H, n_V) = (0, +1)$, intensity modulation is observed along the horizontal and vertical orientations, respectively, and a fork-shaped pattern appears at the center. Meanwhile, for $(n_H, n_V) = (+1, \pm 1)$, modulation occurs along the diagonal diffraction, although a fork-shaped pattern is difficult to discern, even for $(n_H, n_V) = (+1, +1)$.

Spatial frequency filtering was performed for these diffractions. $A(\rho _{fn}, \phi _{fn})$ and the phase distributions for the $nb^{\prime } = 0, \pm 1,+2$ diffraction waves are presented in the images in the first and second rows of Fig. 2b. We obtained a single spiral phase distribution for $nb^{\prime } = \pm 1$, and the rotation angle was reversed depending on the sign of $nb^{\prime }$. Notably, a clear double spiral phase distribution was obtained for the $nb^{\prime } = +2$ diffraction despite the obscure interference intensity pattern; by contrast, a concentric circle pattern was obtained for $nb^{\prime } = 0$. Simulated phase distributions for these Bragg diffraction waves are shown in the figures in the third row of Fig. 2b and are in agreement with the experimental results. We also calculated the TC spectrum for these waves using Eq. (9); the results are shown in Fig. 2c,d. Well-defined peaks are observed at $\ell = \pm 1$ for $nb^{\prime } = \pm 1$ and at $\ell = 0$ for $nb^{\prime } = 0$ waves. For $nb^{\prime } = +2$, contamination of the $\ell = 0$ mode to $\ell = +2$ mode is confirmed, similar to the diffraction wave from the $b = 2$ fork grating. (We also simulated inline holography experiments applied for a grating constructed from two fork gratings with different pitches, as presented in Supplemental Material^27^).

**Figure 3:**
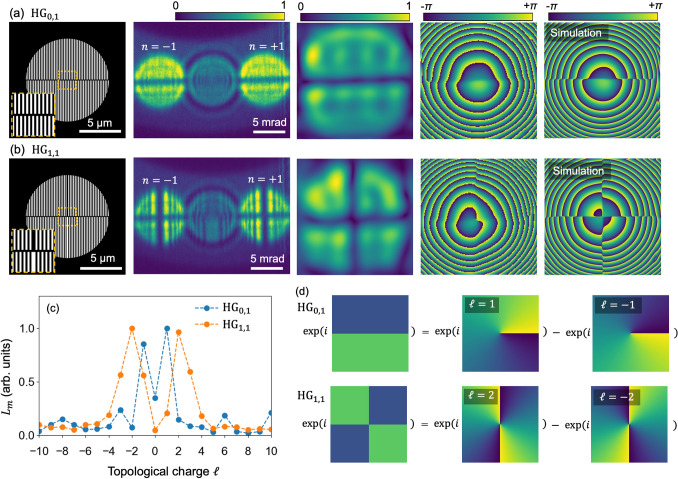
(**a**,**b**) Inline holography for Hermite–Gauss states $\hbox {HG}_{0,1}$ and $\hbox {HG}_{1,1}$. Schematic of the gratings for producing $\hbox {HG}_{0,1}$ and $\hbox {HG}_{1,1}$ states (first column). Insets show enlarged views around the center of the gratings. holographic images for the diffraction waves from these gratings (second column), $A(\rho _{fn}, \phi _{fn})$ (third column) and the phase distribution (fourth column) for the $n = +1$ diffraction, and the simulated phase distribution (fifth column). (**c**) TC spectra for the $\hbox {HG}_{0,1}$ and $\hbox {HG}_{1,1}$ states; figure (**d**) shows that these states can be decomposed into two LG states.

**Figure 4:**
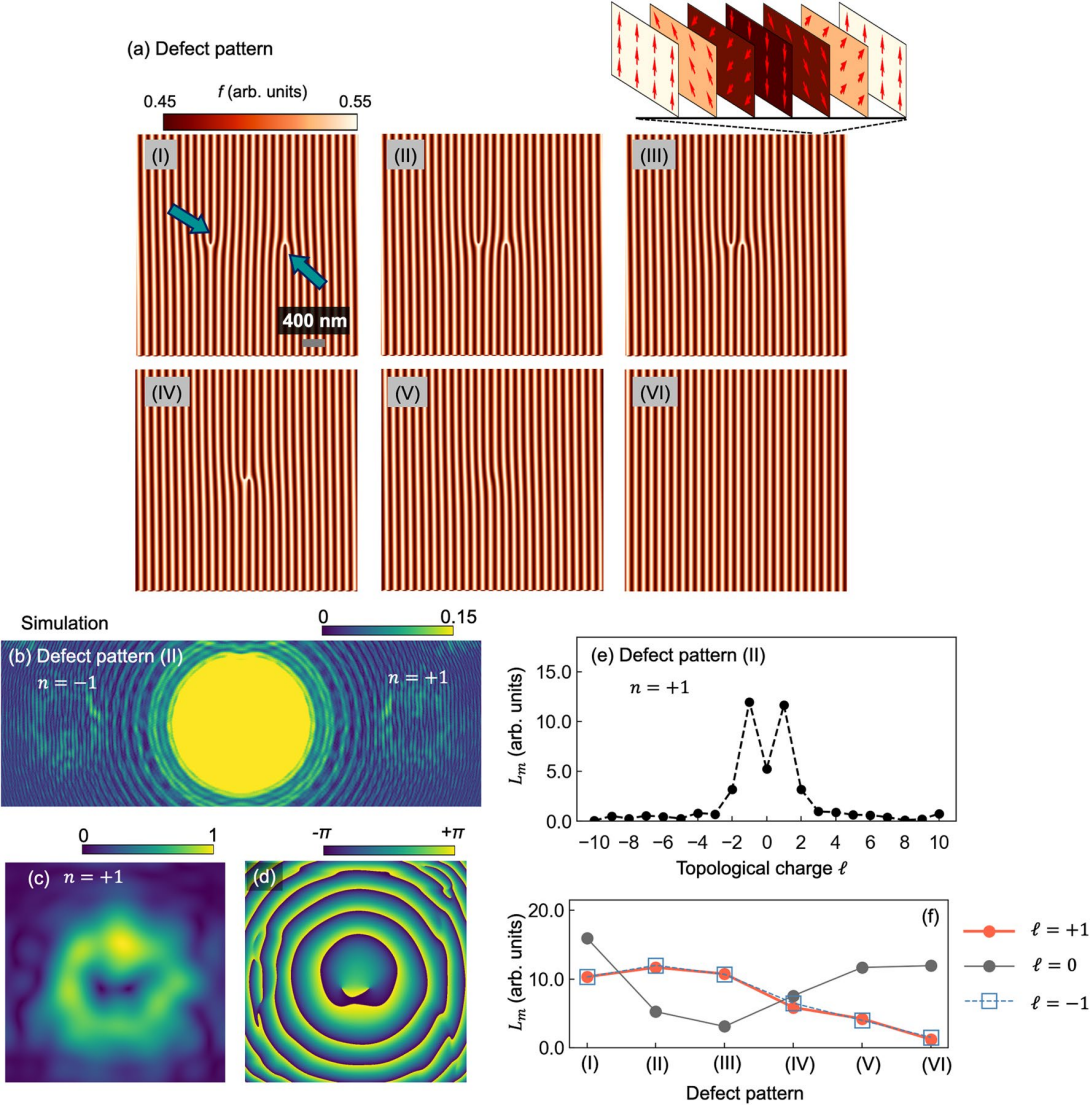
Simulation of inline holography for pair annihilation of edge dislocations in a helical magnetic texture. (**a**) (I)–(VI) Schematic of pair annihilation of the defects. Positive and negative edge dislocations (indicated by arrows in figure (I)) annihilate when they meet. (**b**) Holographic pattern calculated for $n = \pm 1$ diffraction from defect pattern II. (**c**) $A(\rho _{fn}, \phi _{fn})$, (**d**) phase distribution, and (**e**) TC spectrum for the $n = +1$ diffraction wave. (**f**) $L_m$ of $\ell = \pm 1, 0$ for the $n = +1$ diffraction wave generated from each defect pattern.

The present technique was also applied to Hermite–Gaussian ($\hbox {HG}_{u,v}$) modes. The gratings producing $\hbox {HG}_{0,1}$ and $\hbox {HG}_{1,1}$ states are illustrated in the images in the first column of Fig. 3a,b. Holographic images obtained for the diffraction waves from these gratings are shown in the images in the second column, where a horizontal intensity modulation appears for each wave. For both waves, stripes in the upper half of the diffraction pattern are out of phase with those in the lower half by $\pi$. We extracted $A(\rho _{fn}, \phi _{fn})$ and the phase distributions for the $n = +1$ order diffraction waves, as shown in the images in the third and fourth columns. We also simulated the phase distribution for these waves, as shown in images in the fifth column. The results were found to reasonably reproduce the experimental results. These results confirmed that phase inversion occurs at the horizontal center line for the $\hbox {HG}_{0,1}$ mode and at the horizontal and vertical center lines for the $\hbox {HG}_{1,1}$ mode. Figure 3c shows the TC spectra for the $\hbox {HG}_{0,1}$ and $\hbox {HG}_{1,1}$ waves. Well-defined peaks are observed at $\ell = \pm 1$ and $\ell = \pm 2$ for $\hbox {HG}_{0,1}$ and $\hbox {HG}_{1,1}$, respectively, indicating that $\hbox {HG}_{0,1}$ ($\hbox {HG}_{1,1}$) mode can be decomposed into $\ell = \pm 1$ ($\ell = \pm 2$) LG modes as shown in Fig. 3d. It is known that transforming an HG mode to a LG mode and vice versa are possible^23^. These results imply that our proposed technique is also effective for HG waves as well as for soft X-ray waves produced from arbitrary defects.

## Simulation of inline holography for dynamics of topological defects

Finally, we propose a practical application of our inline holography measurement to characterize the dynamics of topological defects in a magnet. Topological defects, such as edge dislocations (as shown in Fig. 4a), have been commonly observed in several magnetic textures, including ferromagnetic stripe, helical, and skyrmion domain structures. Previous studies have reported the dynamics of topological defects in these magnetic textures under thermal fluctuation or a magnetic field^17,28–30^.

Here, we simulated an inline holography experiment for pair annihilation of magnetic edge dislocations in a helical magnetic lattice, which is suggested by the calculation in Ref.^30^. Figure 4a illustrates positive and negative edge dislocations (with topological numbers $b = \pm 1$) in a helical magnetic lattice with a pitch of 120 nm. These two defects annihilate each other when they meet. We set the X-ray absorption to $f_{\uparrow } = 0.55$ for the magnetic moments pointing upward and to $f_{\downarrow } = 0.45$ for those pointing downward so that the XMCD signal $(f_{\uparrow } - f_{\downarrow } )/ (f_{\uparrow } + f_{\downarrow } )$ was comparable to the typical XMCD value for a transition-metal magnet at the $L_{\mathrm{III}}$ absorption edge (i.e., $(f_{\uparrow } - f_{\downarrow } )/ (f_{\uparrow } + f_{\downarrow } ) = 0.1$). For the outside of the sample, the X-ray absorption was set to $f_{\mathrm{outside}} = 0.02$. The simulated experimental geometry is the same as that shown in Fig. 1a, where the samples are placed at $z_1 = 600\ \upmu$m from the focal point. Figure 4b shows the calculated holographic image for diffraction waves with $n = \pm 1$ generated from defect pattern II, as obtained using the scaled FFT method^26^. $A(\rho _{fn}, \phi _{fn})$ and the phase distribution for the $n = +1$ diffraction are obtained by spatial frequency filtering, as shown in Fig. 4c,d. The phase distribution clearly contains two singularities around the center, around which the phase locally rotates along the opposite direction, indicating that $\ell = \pm 1$ modes are generated from the positive and negative edge dislocations, respectively. The TC spectrum for the diffraction wave (shown in Fig. 4e) clearly shows that the diffraction wave is decomposed to mainly $\ell = \pm 1$ modes as well as the $\ell = 0$ mode, which reflects the topological number of defect pattern II.

Figure 4f shows the spectrum amplitude $L_m$ of $\ell = 0, \pm 1$ modes for the diffraction wave generated from each defect pattern. When the two defects are far from each other (as shown in Fig. 4a-I), the $L_m$ of $\ell = 0$ mode is large compared with the $L_m$ of the $\ell = \pm 1$ modes. As the defects approach the center, rotational phase components of the magnetic texture increase in the X-ray irradiation area, which results in an increase of the $\ell = \pm 1$ modes and a decrease of the $\ell = 0$ mode of the diffraction wave. As they approach further, the $\ell = 0$ mode increases again, with a concomitant decrease of the $\ell = \pm 1$ modes, reflecting the cancellation of the phase components of the $b = \pm 1$ defects. Finally, only the $\ell = 0$ mode wave is generated when the defects totally disappear. The dependence shown in Fig. 4f represents the changes of the topological number on the magnetic texture associated with pair annihilation of the topological defects. Thus, acquiring the changes of the spectrum amplitude $L_m$ enables the dynamics of topological defects in magnetic textures to be investigated.

## Summary

We demonstrated inline holography experiments for soft X-ray vortex beams produced by several types of optical gratings. After analyzing the obtained holographic images, the TC of the LG vortex waves is successfully extracted, which reflect the topological numbers of the gratings, as well as the HG waves. In addition, we presented a simulation of the practical application of inline holography technique for pair annihilation of magnetic edge dislocations in a helical magnetic lattice. These results imply that the present technique can be used as an effective probe for the dynamics of topological defects on several types of magnetic textures. Furthermore the present technique has the potential to be applied for measurements of fast generation and control of skyrmion or chiral soliton by irradiating vortex beams as suggested in recent theoretical approaches^4–6^.

## Methods

Soft X-ray inline holography experiments were performed at beamlines BL-13A and BL-16A of the Photon Factory, KEK, Japan. The incident X-rays were focused via a FZP with outer and center beam-stopper radii of 60 $\upmu$m and 30 $\upmu$m, respectively. The X-ray wavelength was tuned at $\lambda$ = 1.6 nm, which ideally resulted in a focal length of 1.5 mm. The first order diffraction from the FZP was selected by an OSA with a radius of 5 $\upmu$m positioned in the vicinity of the focal point. Interference patterns were recorded using an in-vacuum CCD camera (Teledyne Princeton PMI2048, 2048 $\times$ 2048 pixels, pixel size 13 $\upmu$m). Fork gratings used in the present study were made from Ta metal with a thickness of 300 nm deposited onto a membrane of $\hbox {Si}_3\hbox {N}_4$.

## Supplementary Information

Supplementary Information.
