# Supplementary material for: Topological charge of soft X-ray vortex beam determined by inline holography
Source: Sci Rep. 2022 Jan 20;12:1044. doi: 10.1038/s41598-022-04933-5 (PMC8776782; doi:10.1038/s41598-022-04933-5)
Supplement: Supplementary file 1 — Supplementary Information. [file 41598_2022_4933_MOESM1_ESM.pdf]

Supplemental Material : Topological charge of soft X-ray vortex beam determined by inline holography

Yuta Ishii[1], Hironori Nakao[2], Masaichiro Mizumaki[3], Yusuke Wakabayashi[1], Taka-hisa Arima[4], and Yuichi Yamasaki[2,4,5]

[1]Department of Physics, Tohoku University, Sendai 980-8578, Japan
[2]Photon Factory, Institute of Materials Structure Science, High Energy Accelerator Research Organization, Tsukuba 305-0801, Japan
[3]Japan Synchrotron Radiation Research Institute (JASRI/SPring-8), Sayo 679-5198, Japan
[4]RIKEN Center for Emergent Matter Science (CEMS), Wako 351-0198, Japan
[5]Research and Services Division of Materials Data and Integrated System (MaDIS), National Institute for Materials Science (NIMS), Tsukuba, 305-0047, Japan

S1. Simulation of inline holography for a fork grating constructed from two fork gratings with different pitches.

In the main text, we demonstrated an inline holography experiment for a grating constructed from two perpendicularly oriented fork gratings with same pitch expressed as Eq. (10). Here we present a simulation of inline holography experiments applied for a grating constructed from two fork gratings with different pitches. The transmission of this grating is expressed as,

$$t(\rho,\phi) = \frac{1}{2}\left(1+\mathrm{sgn}\left[\sin\left(\frac{2\pi}{d_H}\rho\cos\phi + b_H\phi\right)\right]\right) \times \frac{1}{2}\left(1+\mathrm{sgn}\left[\sin\left(\frac{2\pi}{d_V}\rho\sin\phi + b_V\phi\right)\right]\right), \tag{1}$$

where we assumed $b_H = 1$ and $d_H = 150$ nm, and $b_V = 1$ and $d_V = 200$ nm, for a horizontal and a vertical lattices, respectively.

Schematic of the grating is shown in Fig. S1 (a). Hologram pattern of the diffraction waves were simulated using the scaled fast Fourier transform method, where experimental geometry is assumed to be the same as that shown in Fig. 1 (c) in the main text. Figure S1 (b) shows calculated hologram pattern. The diffractions are distributed over a rectangle lattice, and the periods of the interference modulation are different between horizontal and vertical diffraction waves. These results are attributed to the facts that the diffraction peak position and the period of interference intensity modulation are dependent on the pitches of the grating (the details are found in Ref. [24]).

Meanwhile, in common with the experiments demonstrated in the main text, generated vortex waves are ideally expressed by TCs described as $\ell = nb' = n_H b_H + n_V b_V$, where n_H and n_V are the order of the Bragg diffraction along the horizontal and vertical directions, respectively. Figure S1 (c) shows a phase distribution of each diffraction wave obtained by means of spatial frequency filtering. We obtained single spiral phases for $nb' = \pm 1$, a double spiral phase for $nb' = 2$, and a concentric circle phase for $nb' = 0$ diffractions. These results are same as the case of the grating constructed from two gratings with the same d values, which is demonstrated in the main text.

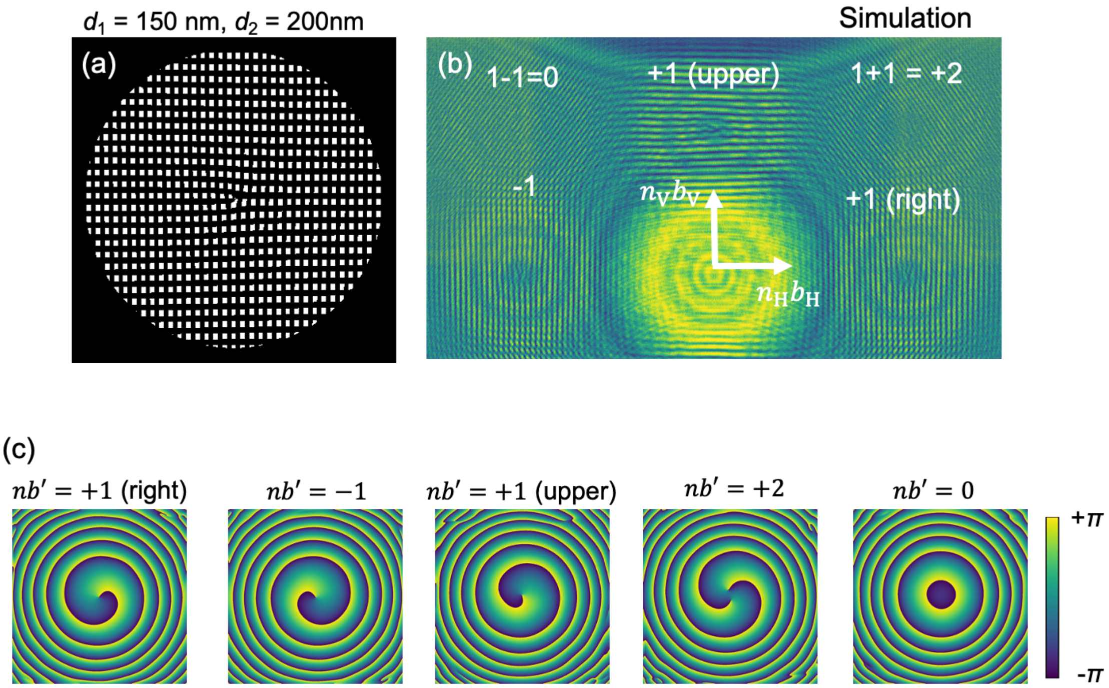

Figure S1: Simulation of inline holography for diffraction waves from a grating constructed from two fork gratings with different d values. (a) Schematic view of the grating. (b) Hologram image of the diffraction waves from the grating. (c) Phase distribution for each diffraction wave obtained by spatial frequency filtering.
